# Electrochemical and photoluminescence response of laser-induced graphene/electrodeposited ZnO composites

**DOI:** 10.1038/s41598-021-96305-8

**Published:** 2021-08-25

**Authors:** N. F. Santos, J. Rodrigues, S. O. Pereira, A. J. S. Fernandes, T. Monteiro, F. M. Costa

**Affiliations:** grid.7311.4 0000000123236065 I3N, Department of Physics, University of Aveiro, Campus de Santiago, 3810-193 Aveiro, Portugal

**Keywords:** Graphene, Nanowires, Structural properties, Synthesis and processing, Batteries, Electrochemistry, Photocatalysis, Porous materials, Chemical physics, Optical spectroscopy

## Abstract

The inherent scalability, low production cost and mechanical flexibility of laser-induced graphene (LIG) combined with its high electrical conductivity, hierarchical porosity and large surface area are appealing characteristics for many applications. Still, other materials can be combined with LIG to provide added functionalities and enhanced performance. This work exploits the most adequate electrodeposition parameters to produce LIG/ZnO nanocomposites. Low-temperature pulsed electrodeposition allowed the conformal and controlled deposition of ZnO rods deep inside the LIG pores whilst maintaining its inherent porosity, which constitute fundamental advances regarding other methods for LIG/ZnO composite production. Compared to bare LIG, the composites more than doubled electrode capacitance up to 1.41 mF cm^−2^ in 1 M KCl, while maintaining long-term cycle stability, low ohmic losses and swift electron transfer. The composites also display a luminescence band peaked at the orange/red spectral region, with the main excitation maxima at ~ 3.33 eV matching the expected for the ZnO bandgap at room temperature. A pronounced sub-bandgap tail of states with an onset absorption near 3.07 eV indicates a high amount of defect states, namely surface-related defects. This work shows that these environmentally sustainable multifunctional nanocomposites are valid alternatives for supercapacitors, electrochemical/optical biosensors and photocatalytic/photoelectrochemical devices.

## Introduction

Laser-induced graphene (LIG) and related composites have been studied in a wide range of applications, namely supercapacitors^1–3^, photodetectors^4^, sensors, including gas, piezo and biosensors^5–10^, and catalysis^11,12^, among others. In terms of synthesis, LIG presents several advantages compared to other graphene types and their derivatives, including simplicity, cost-effectiveness, and swiftness of synthesis, without the need for aggressive chemical conditions, extreme chamber temperatures or vacuum. Moreover, LIG can be formed using a wide range of carbon-containing precursors, from graphene oxide to polyimide (PI) and even wood or food^1,2,13,14^. Combined with relatively low sheet resistances (e.g. tens of Ω square^−1^^1^), the edgy and high specific area (e.g. 148.4 m^2^ g^−1^^15^ or 342 m^2^ g^−1^^1^) provided by porous LIG is interesting for all the above-mentioned applications. Additionally, LIG on polymeric substrates such as PI is inherently flexible, promoting its applicability in e.g. lab-on-skin sensing devices or supercapacitors for flexible electronics^1,3,16^. Finally, patterns having sizes down to a few tens of micrometers can be directly formed on the PI via laser beam scanning in the *xy* plane (direct laser writing, DLW) without the need for standard lithography processes, a major advantage regarding the simple and low-cost production of miniaturized devices.

Many efforts have been employed to combine graphene with environmentally sustainable transition metal oxides and wide bandgap semiconductors, among which zinc oxide (ZnO), with an energy bandgap of ~ 3.3 eV at room temperature (RT)^17^. Techniques such as hydrothermal^18^, electrochemical^19–23^, physical vapor deposition^24,25^ and laser-assisted synthesis^26–29^ have been thoroughly explored to produce a wide variety of ZnO crystal morphologies and sizes. The resulting materials can be further mixed with graphene using a myriad of techniques such as drop-casting, dip-coating, and others^30,31^. Additionally, simultaneous synthesis approaches have also been recently attempted^32,33^. Despite its merits, the controlled simultaneous synthesis of such composites is not straightforward and alternative routes to synthesize these composites are still desirable.

The usage of LIG as a substrate for ZnO growth is conceptually appealing. Due to the LIG morphological characteristics, it can potentially allow a superior loading of ZnO structures, which in turn can lead to improved performance in several applications, including supercapacitors^34–36^. High loadings of ZnO in porous graphene could allow for superior specific capacitance, via electric double layer and/or pseudocapacitance. Fast charge/discharge times are promoted by the highly conductive graphene framework and by the swift pseudocapacitive kinetics of transition metal oxides^35^. The high conductivity provided by graphene is also important in improving the electrochemical stability of the transition metal oxides^35^. On the other hand, LIG/ZnO composites can also enhance the actual area for biorecognition element loading in electrochemical biosensors, which combined with the ZnO surface isoelectric point at pH 9–9.5^37^ makes these composites particularly appealing for adsorption-based biorecognition element immobilization. Moreover, the integration of ZnO provides optical properties suitable for exploring combined optical/electrochemical biotransduction or photoelectrochemical sensing, which can provide extended detection ranges, superior sensitivities and/or lower limits of detection^31,38^. ZnO is also a very appealing material for photocatalytic applications owing to its high photosensitivity, stability and bandgap in the near-UV region, being even considered a potential alternative to current benchmark TiO_2_^39^. The increase of the active surface area of ZnO by growing it in a highly porous substrate such as LIG is expected to be profitable in photocatalysis by enhancement of dye adsorption and UV light absorption. Furthermore, having a free-standing photocatalyst is advantageous as it facilitates its removal from the reaction media, which is frequently an issue with the traditional powder materials, and even allowing its re-use.

However, the successful application of these composites is dependent on the quality and characteristics of the LIG/ZnO interface and whether the desired electrochemical and/or optical properties are achieved. Hence, the synthesis and characterization of these composites must be profoundly understood in order to achieve reproducible properties. A yet unexplored synthesis approach is the pulsed electrodeposition of ZnO on LIG. A variety of ZnO morphologies can be obtained at low temperatures via this approach, in a versatile, cost-effective and scalable manner^19–23^. The ZnO electrodeposition is based on the reduction of oxygen-containing precursors in the presence of ionic Zn at the working electrode of an electrochemical cell, via the application of a suitable electric potential. Pulsed electrodeposition, where the potential or current is alternated between deposition/resting status, provides several advantages compared to the static potential (continuous) process, such as renewal of diffusing layer, promotion of ZnO nucleation and the possibility to control the growth via the employed duty cycle and pulse duration or frequency^22,29,40^.

This work explores a wide range of parameters envisaging uniform, conformal and well-intercalated electrodeposition of ZnO rods onto LIG, and describes the morphological, structural, electrochemical and luminescent properties of these novel, environmentally sustainable composites.

## Results and discussion

### Synthesis, morphology and structure

**Figure 1:**
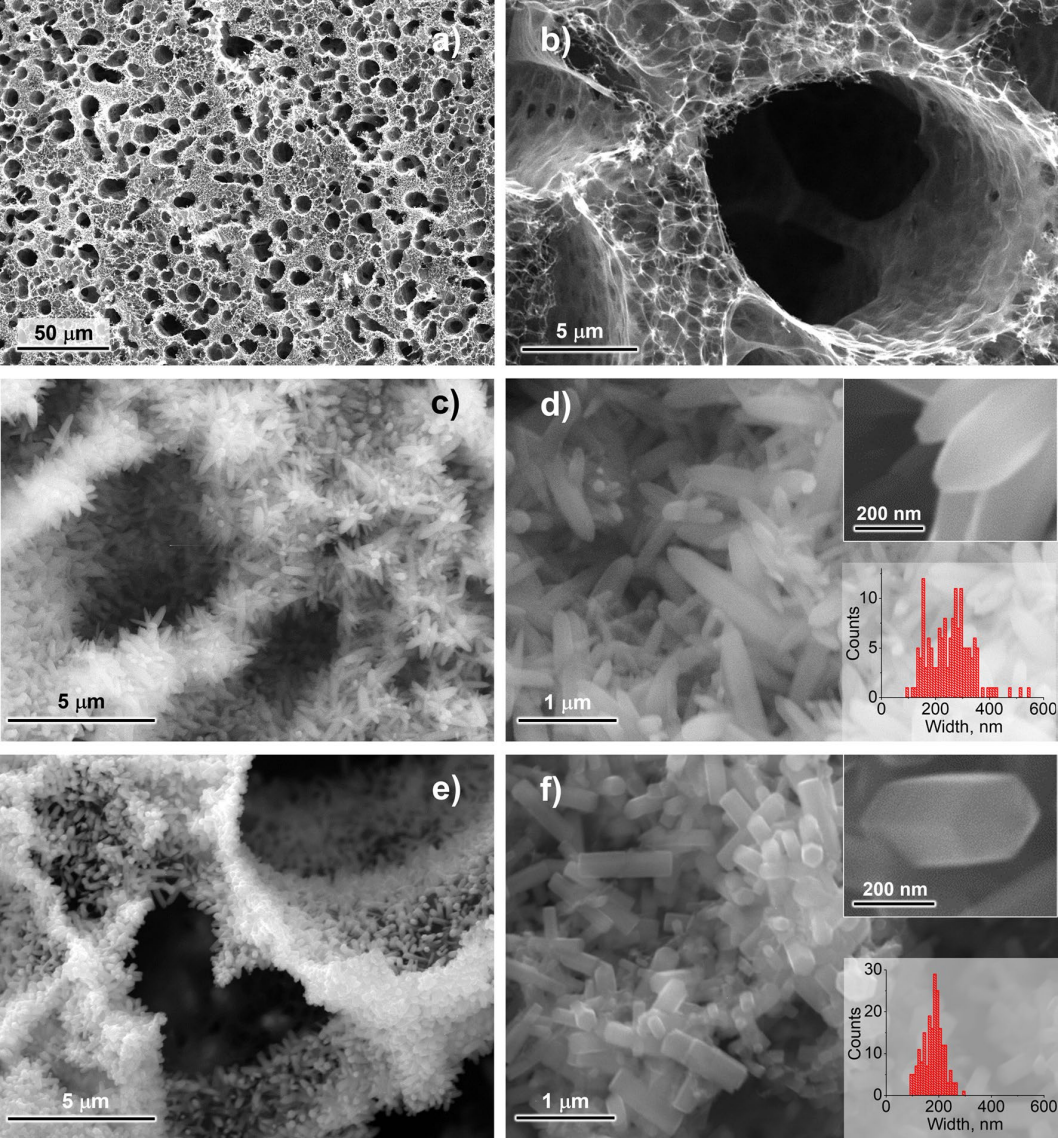
Top-view SEM micrographs of: (**a,b**) bare LIG; (**c,d**) LIG/ZnO after continuous electrodeposition (profile A); (**e,f**) LIG/ZnO after pulsed electrodeposition (profile B). Insets are statistical distribution of rod widths.

The DLW of LIG on PI sheets was accomplished using the parameters listed in Supplementary Table S1. The morphology of the obtained LIG is shown in the SEM images of Fig. 1a,b. One can observe the existence of interconnected flakes in a maze-like 3D arrangement characterized by hierarchical porosity, a well-known morphology for this type of graphene^1,2,6,41,42^. The pores cover a considerable range of sizes, from a few nanometers to a few micrometers, and originate from the gas outrush during the high-power CO_2_ laser beam irradiation of the PI foil. Afterward, LIG served as substrate for ZnO electrodeposition employing the parameters conveniently arranged in Table 1. More information on the ZnO electrodeposition procedure can be found in the “Methods” section and in the Supplementary Material file.

**Table 1:**
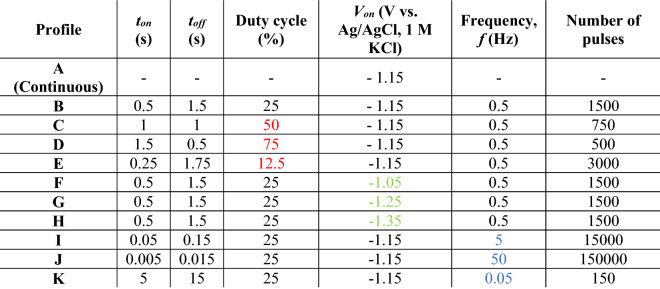
Employed parameters for ZnO electrodeposition on LIG.

**Figure 2:**
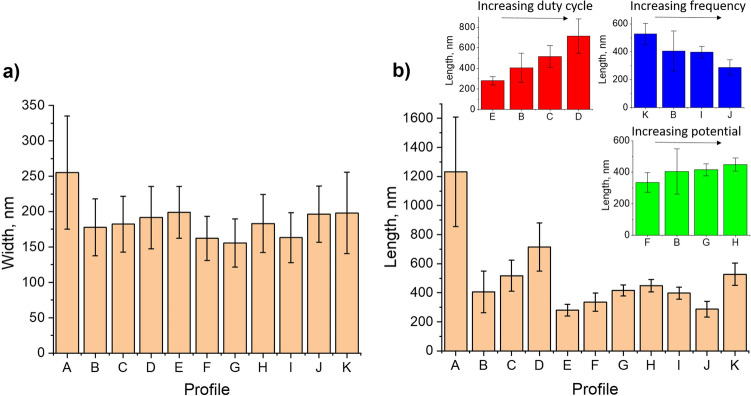
Statistical analysis of ZnO rod (**a**) widths and (**b**) lengths. See Table 1 for complete information on deposition profiles.

Figure 1c–f present the SEM images of the LIG/ZnO composites produced via continuous and pulsed electrodeposition (profiles A and B, respectively). Supplementary Fig. S4 further gathers the SEM images of all the remaining composites produced in this work and Fig. 2 shows the statistical analysis of rod widths and lengths for all profiles. In general, ZnO electrodeposition takes place deep inside the LIG pores and covers its entire surface, whilst maintaining the characteristic porous nature of the 3D-like graphene foam. These constitute fundamental advantages compared to other techniques for ZnO-on-LIG composite production, such as physical vapor deposition, drop casting and dip coating.

The parameter range for the production of ZnO rods on LIG is wide. Despite differences in rod vertical growth, all the pulsed profiles resulted in the production of faceted hexagonal ZnO rods without a preferential orientation, such as the ones in Fig. 1f, independently of the parameters employed. On the contrary, for the continuous process, the rods tend to be more irregular and present a generalized pointy tip morphology (Fig. 1d). The rod average width and respective width distributions are statistically similar for all pulsed profiles (Fig. 2a), while for the continuous profile A rods tend to be wider and with a broader distribution, with a higher tendency for agglomeration. This is indicative that seed dimensions are of utmost importance in defining rod widths in pulsed electrodeposition, but that is overcome in the less controllable, higher deposition rate continuous process.

The pulsed electrodeposition profiles have a relatively small deposition step duration, t_on_, leading to mass transfer-limited growth because the diffusion layer does not have time to expand significantly into the solution^43^. Therefore, a thinner, sub-developed diffusion layer is formed at higher frequencies and a rapid reactant depletion near the electrode surface occurs, explaining the limited ZnO rod vertical growth of profile J (Fig. 2b). In contrast, continuous profile A does not impose such a restriction, leading to an augmented vertical growth of the rods and a thicker ZnO layer. Indeed, the obtained statistics for rod length clearly differentiate the continuous profile A from the pulsed ones (B to K). This is qualitatively supported by the Raman spectra, as described later in this section.

Related literature refers that as long as the resting step duration t_off_ remains approximately equal or higher than t_on_ (50% duty cycle or lower) a complete renewal of the diffusion layer is attained so that the initial surface concentrations of Zn^2+^ and NO^3−^ are completely regenerated^40^. According to this, profile D does not guarantee a proper renewal, and deposition should begin resembling more that of a continuous process in terms of ZnO morphology. An increased vertical growth trend is observed with the increasing percentage of duty cycle (E-B-C-D in Fig. 2b), yet still far from profile A. Besides, a prevalence of faceted hexagonal rod morphologies is maintained employing profile D, contrasting to the dominant pointy tip morphology observed for profile A.

The influence of the deposition potential V_on_ is contemplated by the profiles F, B, G, and H. Again, for all of them it was possible to produce faceted hexagonal ZnO rods. Still, profile F with a V_on_ of − 1.05 V vs. Ag/AgCl denotes a statistically diminished rod growth compared to the remaining ones, as well as a markedly non-homogeneous nature. In fact, it is possible to observe large sample regions where ZnO seeds from pre-treatment did not develop into rods (Supplementary Fig. S4d, top left inset). This effect disappears for profiles employing more negative potentials, indicating that the threshold voltage for sustained ZnO crystal growth reaction is only attained at about − 1.15 V vs. Ag/AgCl (1 M KCl). This is in qualitative agreement with previous findings regarding the ZnO growth rate dependency on the applied potential for continuous electrodeposition^23^.

**Figure 3:**
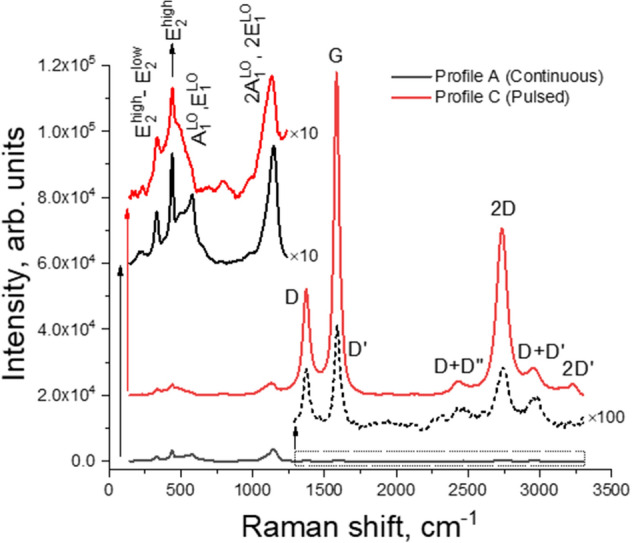
Background-subtracted μ-Raman spectra at 442 nm photon excitation of the LIG/ZnO composites produced by continuous (profile A, solid black line) and pulsed (profile C, solid red line) ZnO electrodeposition. The spectra are normalized to the peak intensity of the E_2_^high^ vibrational mode of ZnO (~ 439 cm^−1^) and shifted in intensity for clarity.

Figure 3 shows the μ-Raman spectra of profiles A and C acquired at 442 nm laser excitation wavelength. The LIG-related phonon modes are found in the range from ~ 1250 to 3500 cm^−1^, resembling those typically observed in rGO^44,45^. The D-band is peaked at 1371 cm^−1^ and is associated with the sp^2^ coordinated defective/disordered carbon phases^44^. The most intense mode appears at ~ 1587 cm^−1^, corresponding to the G-band, which is related to C–C bond stretching in sp^2^ hybridizations^46^. This band is accompanied by a small shoulder at ~ 1620 cm^−1^, the D′ band, another defect-activated vibrational mode. The D′′, a very weak band placed at ∼ 1100 cm^−1^ that appears combined with the D band at ~ 2450 cm^−1^, is assigned to vibrational modes from the LA branch^47^. Additionally, other modes associated with multi-phonon processes are visible, namely, D + D′′, 2D, D + D′ and 2D′ modes, commonly present in the spectra of graphene materials.

The presence of a single, strong and symmetric 2D overtone at 2737 cm^−1^ clearly evidences a graphene-based material and the fact that I_2D_/I_G_ intensity ratio is less than unity points to the formation of multi-layer graphene. The I_D_/I_G_ intensity ratio constitutes a measure of LIG’s lattice defect density and is seen to fluctuate among all probed samples. This is mainly caused by an inherent statistical distribution within each LIG sample^6^.

The typical Raman active vibrational modes of the ZnO wurtzite crystalline structure can be identified in the frequency range from ~ 200 to 1200 cm^−1^. Three peaks can be clearly observed in this region for all samples, corresponding to the E_2_^high^–E_2_^low^ peaked at ~ 335 cm^−1^, E_2_^high^ at ~ 439 cm^−1^, and the 2A_1_(LO), 2E_1_(LO) placed at ~ 1137–1142 cm^−1^. The E_2_^high^–E_2_^low^ and 2A_1_(LO), 2E_1_(LO) modes are overtones and combined modes related to multi-phonon processes while the others correspond to the first-order Raman scattering at k = 0^48^. The broad peak present in the ~ 570–590 cm^−1^ range is likely to be an overlap of the A_1_(LO) and E_1_(LO) phonon modes which are known to appear in this region^48^. Finally, other features include a weak mode(s) at ~ 210–230 cm^−1^, possibly arising from 2(TA) and/or 2E_2_^low^ processes, a peak at 286 cm^−1^, attributable to B_1_^high^–B_1_^low^ process, and a broad shoulder within the ~ 480–540 cm^−1^ range, possibly related to an overlap of 2(LA) and 2B_1_^low^ overtones^48^.

The continuous profile A originates a lower (higher) intensity and definition of the vibrational modes associated with LIG (ZnO). This can be attributed to the thicker ZnO rod layer formed in this case and the limited penetration depth of the laser beam, probing mostly the ZnO crystals and not the LIG underneath. Likewise, considering the pulsed profiles only, the differences in the relative intensities of ZnO and LIG modes can be explained also by dissimilar ZnO rod growth. Nevertheless, both continuous and pulsed profiles result in similar Raman fingerprints (see Supplementary Fig. S5 for the remaining spectra), despite differences in the relative intensity of ZnO vibrational modes, which was found to be dependent on the probed region, possibly related to local preferential orientation of the rods.

X-ray diffraction (XRD) measurements were also conducted in order to further assess structural information on the composites and the results are shown in Supplementary Fig. S6. Both profile B (pulsed electrodeposition) and profile A (continuous electrodeposition) samples’ diffractograms denote narrow ZnO (wurtzite) peaks with similar full widths at half maximum, e.g. $\Delta \left(2\theta \right)\cong 0.24^\circ$ for the ($10\overline{1 }1$) reflection, showing a similar (high) crystallinity degree for the two samples. This corroborates the observed in the Raman measurements of Fig. 3 and Supplementary Fig. S5. Moreover, no additional ZnO_x_ phases were identified, also in agreement with the Raman spectra.

### Electrochemical response

The morphological and structural characteristics of these LIG/ZnO composite electrodes are conceptually interesting for integrating flexible and efficient supercapacitors as well as versatile and highly sensitive biosensors. Hence, electrochemical measurements were carried out to assess the non-faradaic (capacitive) and faradaic response of the composites.

**Figure 4:**
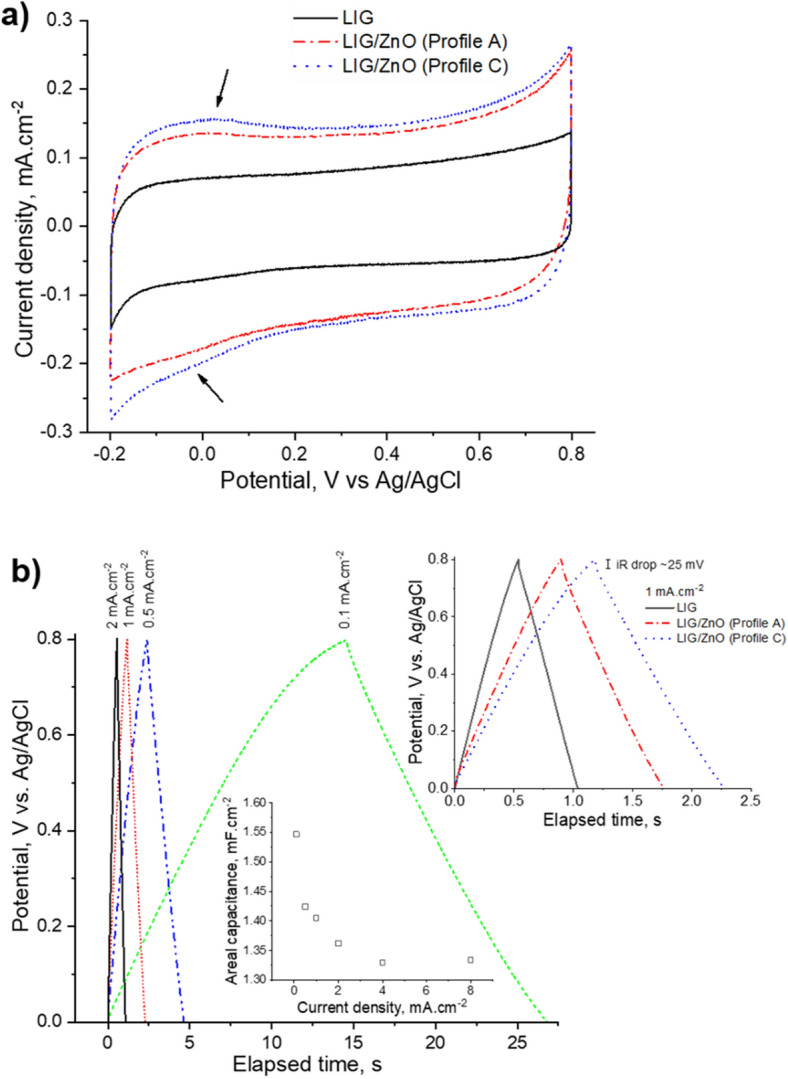
(**a**) Cyclic voltammograms in 1 M KCl aqueous solution. The scan rate is 0.1 V s^−1^. (**b**) GCD curves for the LIG/ZnO (profile C) electrode. The capacitance dependence on the current density (bottom inset) and the comparison with bare LIG and LIG/ZnO (profile A) electrodes (top right inset) are also shown.

In Fig. 4a are depicted the cyclic voltammograms (CV) in 1 M KCl aqueous solution for bare LIG and LIG/ZnO composites, either employing continuous (profile A) or pulsed (profile C) electrodeposition. The CVs maintain a fairly symmetric, quasi-rectangular shape after the ZnO electrodeposition underlining well-behaved capacitive response and low resistive losses, even at fast potential scan rates of 0.1 V s^−1^. The area inside the CVs is larger after ZnO deposition, denoting increased capacitance. The CVs of LIG/ZnO electrodes show pseudocapacitance features around 0 V vs. Ag/AgCl related to hydrogenation of ZnO surface, as identified by the arrows in Fig. 4a.

In Fig. 4b are depicted the galvanostatic charge–discharge (GCD) curves for the same samples within the 0 V to 0.8 V range. The triangular shape of the bare LIG curve denotes little distortion, evidencing nearly ideal electric double layer capacitive behavior (top right inset). For the LIG/ZnO electrodes, the curve shape starts to become distorted due to pseudocapacitive faradaic reactions. The ohmic voltage drop at the beginning of the discharge curves is small for LIG (~ 20 mV at 1 mA cm^−2^) and does not increase significantly after ZnO electrodeposition (30 and 25 mV for profiles A and C, respectively), underlining that good LIG/ZnO interfacial contact is attained. These low internal resistances are an important aspect regarding the efficiency of supercapacitors since the energy dissipated into heat is minimal during device charging and discharging.

The areal capacitance increased from bare LIG to LIG/ZnO (profile A) and to LIG/ZnO (profile C) sequentially (0.645, 1.11, and 1.41 mF cm^−2^, respectively, at 1 mA cm^−2^), see top right inset of Fig. 4. Moreover, LIG/ZnO electrodes have shown superior stability being able to retain about 91.5% of the initial capacitance after 5000 GCD cycles (Supplementary Fig. S7). After an initial decrease of about 6% within the first 100 cycles, the electrode denotes minimal losses after 500 cycles, suggesting that many tens of thousands of cycles are possible without significant deterioration of stored capacity. In addition to the fact that ZnO is chemically stable in neutral to alkaline conditions, these results are promising towards the application of these composites in supercapacitors^34,36^.

**Figure 5:**
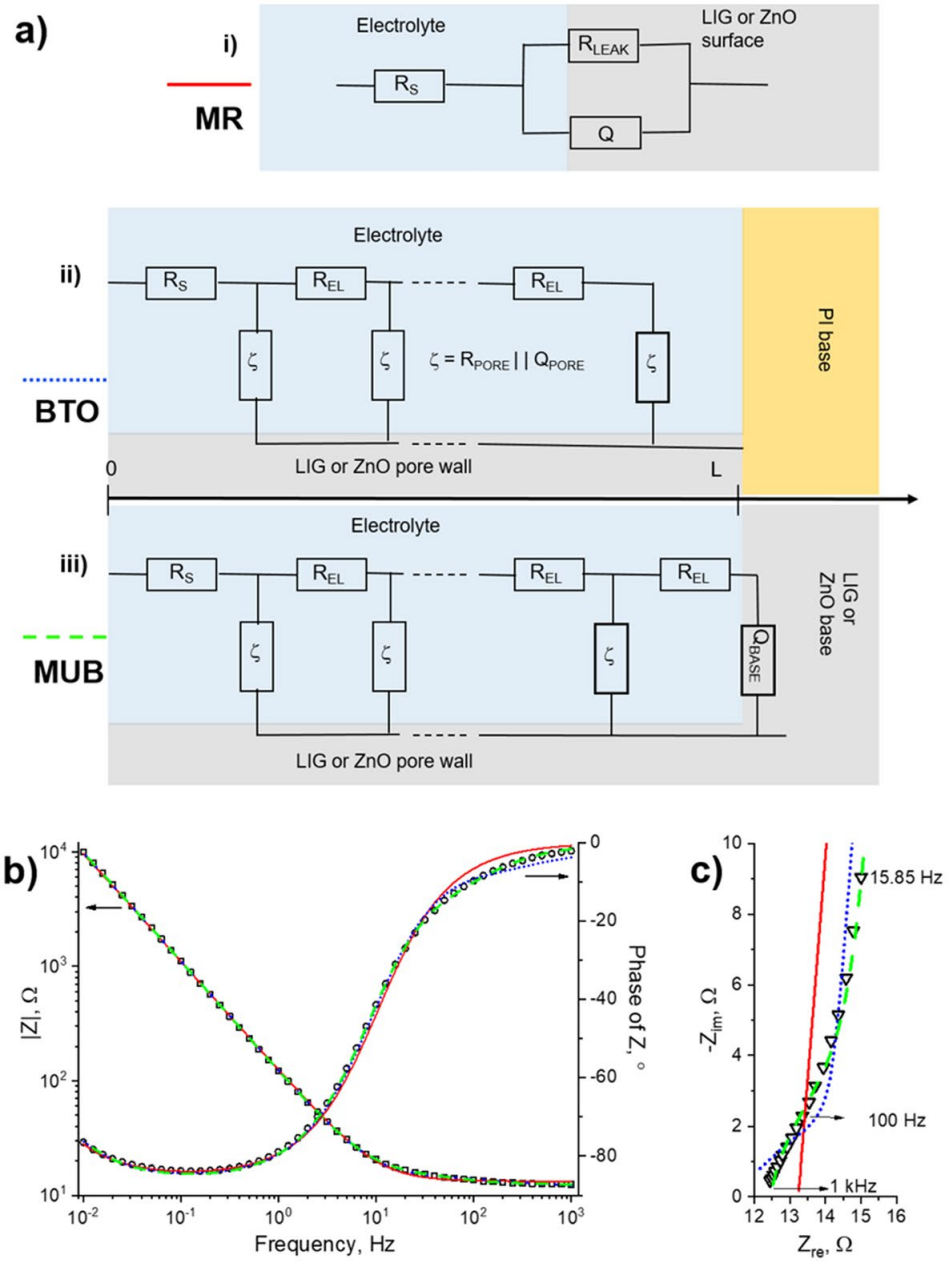
(**a**) Schematics of the equivalent circuits used to model the impedimetric response of LIG and LIG/ZnO electrodes: (i) Modified Randles (MR), (ii) Bisquert open (BTO) and (iii) modified unified Bisquert (MUB). In (ii) and (iii), L represents pore depth and dashed circuit lines represent the stepwise repetition of the R_EL_||ζ blocks along the pore. (**b**) Bode and (**c**) high-frequency portion of the Nyquist plot for the LIG/ZnO (profile C) electrode. Lines are the fittings employing the models as identified in (**a**) at the left side of each circuit. The electrolyte is 1 M KCl aqueous solution.

EIS experiments were conducted in order to better understand the mechanisms beyond the electrochemical response of the different sample types, including the role of porosity. Three distinct equivalent circuits were tested to model the impedimetric response, as schematically shown in Fig. 5a. The modified Randles (MR) circuit (i) comprises a constant phase element Q modelling non-ideal capacitive behavior attributable to surface roughness, an equivalent series resistance, R_s_, which gathers all uncompensated resistances across the cell, and a resistance related to capacitors’ self-discharge (R_LEAK_). The Bode impedance (Z) phase plot of Fig. 5b clearly shows the beginning of a transition from a capacitive to a resistive behavior in the lower frequency portion associated with R_LEAK_. Despite a good correlation at lower frequencies, the MR circuit shows a poor agreement with the impedance spectrum above ~ 1 Hz, as seen in the Bode plots of Fig. 5b and perhaps even more clearly in the Nyquist plot of Fig. 5c. In fact, this simple model cannot cope with the relatively complex (porous) morphology of the LIG electrodes’ surface. Hence, two transmission line models based on the work of Bisquert^49,50^ were also considered, as shown in Fig. 5a (models ii and iii). R_EL_ represents the pore resistance to electrolyte diffusion and ζ the actual reactions occurring at the inner pore surface.

The difference between the models lies in the nature of the base electrode at the bottom of the pores, so that model (ii), herein named Bisquert open (BTO), considers an insulating base (i.e. the base is open-circuited) and model (iii), herein named modified unified Bisquert (MUB), considers a non-insulating, electrochemically active base via the Q_BASE_ element. In the present case, these scenarios correspond to having a PI or LIG/ZnO base electrodes, respectively, as schematically shown in Fig. 5a. It is clear from Fig. 5b that the BTO model (ii) is more effective than the MR one in modelling the transition from the capacitive to the ohmic resistance regimes towards higher frequencies. However, it still is unable to explain the behavior at higher frequencies, see dotted blue line in Fig. 5c. On the contrary, the MUB model (iii) results in excellent agreement in the high-frequency portion (dashed green lines). This is also the case for the bare LIG and LIG/ZnO (continuous profile A) electrodes (see Supplementary Fig. S8).

The fitting parameter values are presented in Supplementary Tables S3–S5 online for models (i), (ii), and (iii), respectively. Given the excellent fitting results of the MUB model, it is apparent that the electrolyte is able to diffuse through the pores of the LIG/ZnO electrodes to reach an electrochemically active base, indicating that ZnO electrodeposits have not led to relevant obstructed porosity, including for the continuous profile A. In fact, fittings employing this model resulted in similar diffusion resistance R_EL_ for all electrodes. This differs fundamentally from other reports in the literature where the impedance spectra of LIG^8^ or LIG/ZnO composite^31^ electrodes were modeled using simple MR circuits. In particular, the impedance spectra of composites^31^ prepared by mixing LIG scraped from the polyimide substrate with ZnO tetrapods produced by laser-assisted flow deposition (LAFD), using organic binders, are well described by such models, suggesting the loss of the original porosity of LIG.

The pre-exponential factor (P_0,PORE_) value of the Q_PORE_ element, related to electrodes’ capacitance, more than doubles after ZnO electrodeposition, in qualitative accordance with the capacitance values derived from GCD measurements. It is also clear that ZnO electrodeposition did not relevantly affect the cell’s ohmic resistance R_s_, only a small increase occurring for the thicker ZnO layer (profile A), also in agreement with the GCD measurements.

**Figure 6:**
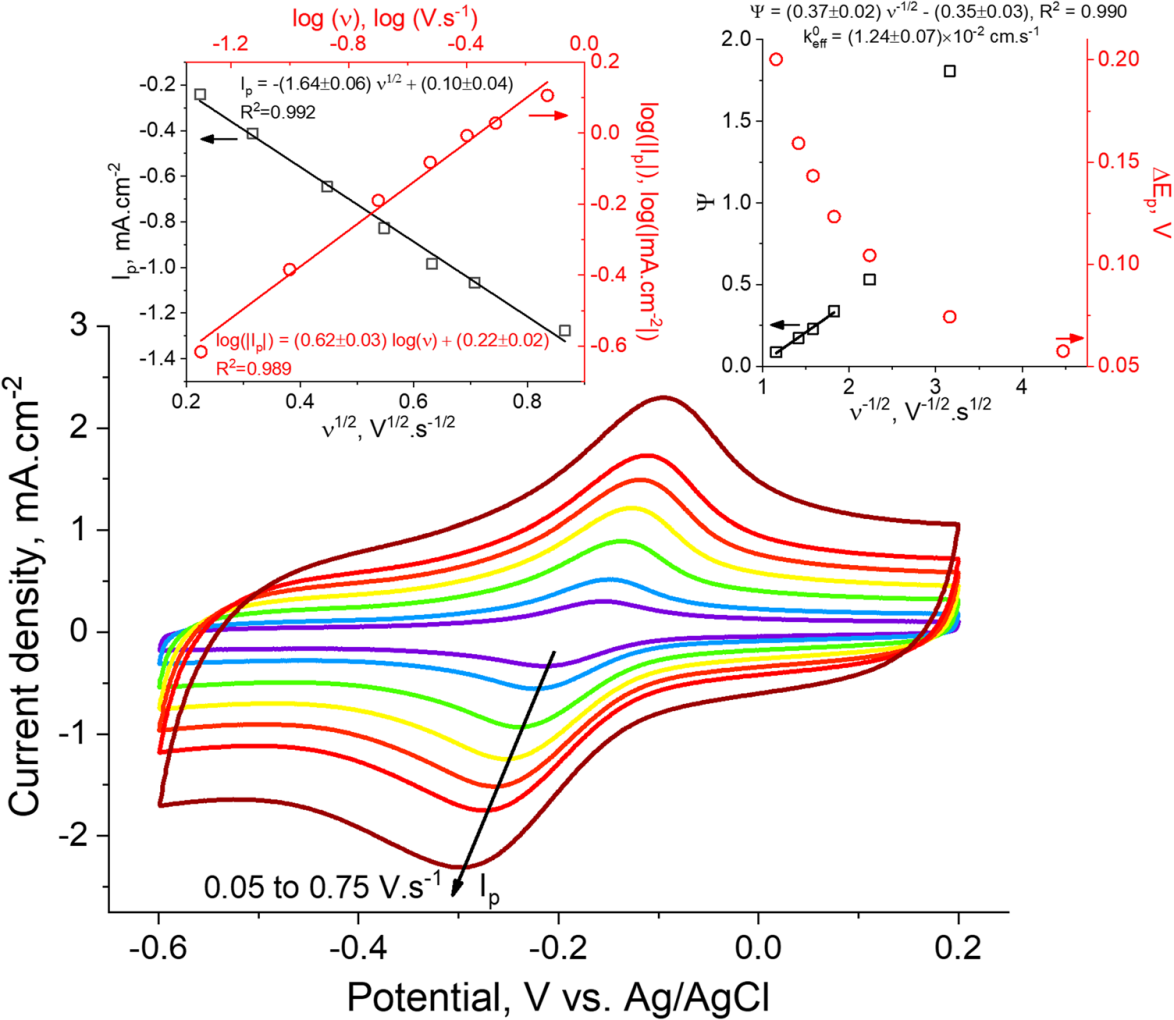
Cyclic voltammograms at varying scan rate $\left( \nu \right)$ for LIG/ZnO (profile C) electrode in aqueous solution of 0.5 mM [Ru(NH_3_)_6_]^3+^ containing 0.1 M KCl. The $\text{log}({\text{I}}_{\text{P}})$−$\text{log}(\nu)$ and ${\text{I}}_{\text{P}}$−${\nu}^{-\frac{1}{2}}$ plots (red circles and black squares, respectively, in the left inset) and the $\Delta {\text{E}}_{\text{p}}({\nu}^{-\frac{1}{2}})$ and the $\Psi ({\nu}^{-\frac{1}{2}})$ (red circles and black squares, respectively, in the right inset) plots are also shown, along with pertinent fittings.

The electron transfer capabilities of the LIG/ZnO composites were assessed via the cyclic voltammograms dependence on the potential scan rate $\left( \nu \right)$, employing 0.5 mM [Ru(NH_3_)_6_]^2+/3+^ (Fig. 6) or 0.5 mM [FeCN_6_]^3−/4−^ (Supplementary Fig. S9) redox probes in 0.1 M KCl aqueous solutions.

As seen in Fig. 6, the voltammograms of [Ru (NH_3_)_6_]^2+/3+^ using LIG/ZnO (profile C) electrode denote well-defined redox waves in a broad scan rate range. In fact, at 50 mV s^−1^ the cathodic/anodic peak-to-peak separation ($\Delta {\text{E}}_{\text{p}}$) is c.a. 57 mV, corresponding to a reversible redox reaction as described by the Nernst equation^51^. For higher scan rates, the electrode enters into a quasi-reversible regime characterized by the departure of $\Delta {\text{E}}_{\text{p}}(\nu)$ values up to c.a. 200 mV at 750 mV s^−1^ (top right inset in Fig. 6). The faradaic peak current densities (${\text{I}}_{\text{P}}$) versus ${\nu}^\frac{1}{2}$ (top left inset) curve follows a well-behaved linear relationship. This indicates that electrochemical reversibility is attained and redox activity is ruled by [Ru(NH_3_)_6_]^2+/3+^ diffusion towards electrode surface in a semi-infinite regime. The absence of relevant adsorption effects is further suggested by the $\text{log}({\text{I}}_{\text{P}})$−$\text{log}(\nu)$ test (top left inset) showing a linear dependence with a slope of about 0.62, relatively close to the theoretical value of 0.5 for a pure semi-infinite diffusion process. Such deviations usually are attributed to weak, reversible adsorption effects^51,52^. On the contrary, limitations to the semi-infinite diffusion due to adsorption and/or the thin layer effect^53^ are clearly observable when employing [FeCN_6_]^3−/4−^ probe, since ${\text{I}}_{\text{P}}$ becomes linearly dependent on and the $\text{log}({\text{I}}_{\text{P}})$−$\text{log}(\nu)$ slope grows to 0.75 (see Supplementary Fig. S9). The heterogeneous electron transfer standard rate constant (${\text{k}}_{\text{eff}}^{0}$) using the [Ru(NH_3_)_6_]^2+/3+^ probe was derived employing the Nicholson method as described in the “Methods” section. A value of ${\text{k}}_{\text{eff}}^{0}\cong 1.24\times {10}^{-2}$ cm s^−1^ was obtained for LIG/ZnO (profile C), indicating swift electron transfer comparable to that of bare LIG^6^ and other reference carbon-based electrodes using similar [Ru(NH_3_)_6_]^2+/3+^ concentration in aqueous solutions^54–56^.

It is thus clear that the LIG/ZnO interface and the ZnO rods layer do not pose a significant resistance barrier to electron transfer, indicating that LIG/ZnO composites are interesting materials for electrochemical sensors. Moreover, as discussed in the next section, ZnO adds other functionalities, such as RT luminescence within the near UV–Vis range, enlarging the scope of application to e.g. simultaneous electrochemical and optical detection of bioanalytes, photoelectrochemical biosensors and photocatalysis-based devices.

### Photoluminescence and photoluminescence excitation

**Figure 7:**
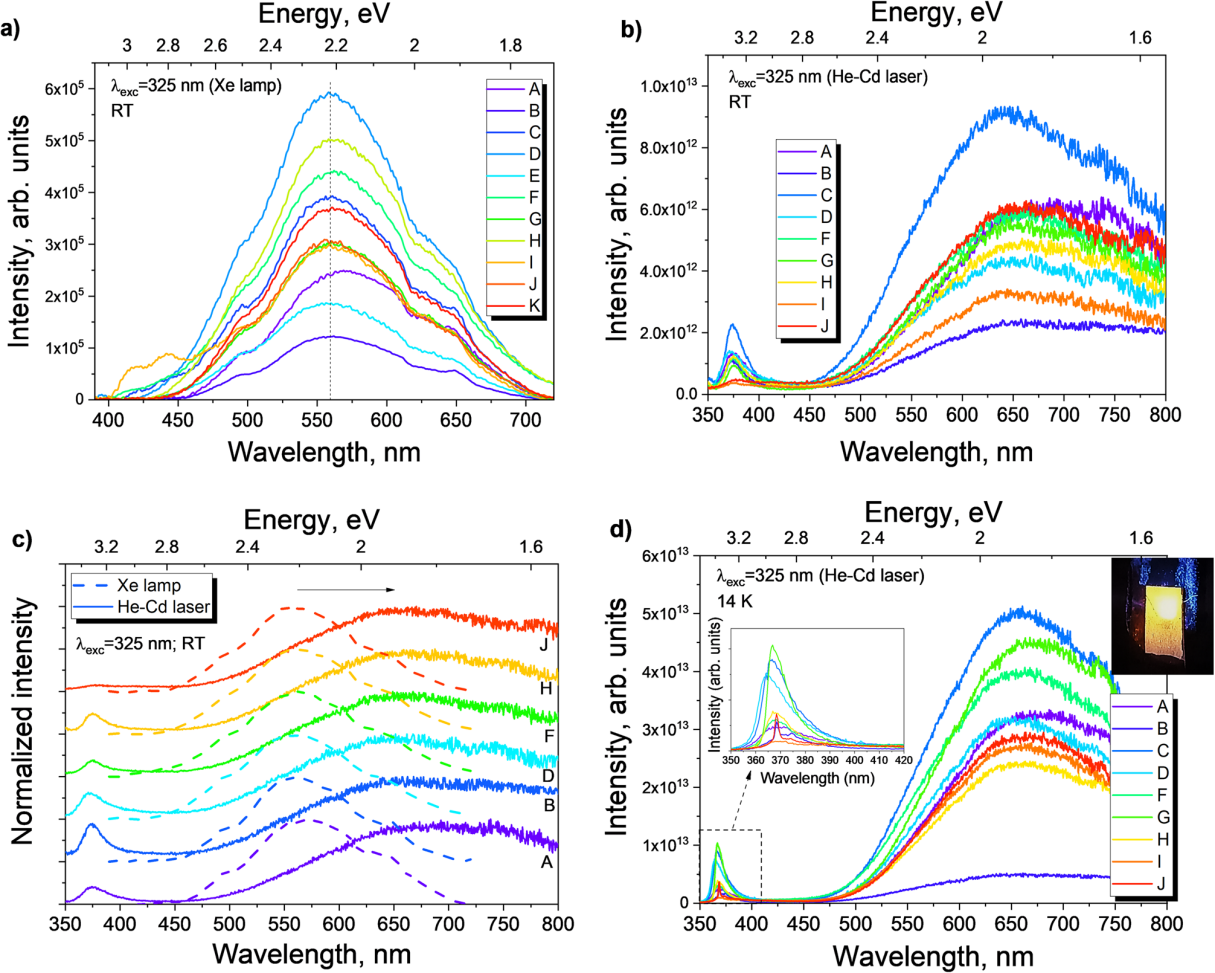
RT PL spectra of (**a**) all composites (profiles A to K) under 325 nm excitation of a Xe lamp and (**b**) selected composites probed with the 325 nm laser line of a He-Cd laser, showing a comparison of their absolute intensity. Note that in (**a**) a longpass filter L38 was employed, thus cutting off the signal below ~ 400 nm. (**c**) Comparison of normalized PL spectra for selected profiles under the same energy excitation (325 nm) but different excitation density conditions (lamp *vs* laser). The spectra were vertically shifted for clarity. (**d**) 14 K PL spectra of selected composites excited with the 325 nm laser line of the He-Cd laser. The insets correspond to an amplification of the UV region and a photograph of the orange/red emission at 14 K for profile C. Profile A corresponds to a continuous electrodeposition and profiles B to K correspond to pulsed electrodeposition. Pulsed electrodeposition profiles have the following relations: Increasing duty cycle (E-B-C-D), increasing (more negative) pulsed potential magnitude (F-B-G-H), and increasing pulse frequency (K-B-I-J). See Table 1 for complete information on profiles.

Figure 7a depicts the PL spectra acquired for all LIG/ZnO composites (profiles A to K) at RT when excited with 325 nm from a Xe lamp, showing that, in all cases, the spectra are dominated by a broad visible band in the orange/red spectral region. The spectral shape and peak position of the emission bands are similar for all composites. Yet, a small redshift of the peak position occurs in the case of profile A (continuous deposition). While the PL bands of the composites prepared by pulsed electrodeposition (profiles B to K) are peaked at ~ 558 nm (~ 2.22 eV), the emission band displayed by LIG/ZnO (profile A) has its maximum at ~ 570 nm (~ 2.17 eV). This is likely to be related to the difference in the synthesis conditions, which may give rise to the formation of different defect centers and/or defect concentration.

In fact, by analysing the PL spectra of selected composites recorded under higher excitation density (He–Cd laser, Fig. 7b), one can clearly note a shift of the peak position of the visible band towards lower energies (longer wavelengths) for all probed composites. Furthermore, a broadening of the band is also observed. Both observations undoubtedly indicate the existence of multiple recombination channels contributing to the overall emission. Such findings can be better observed in Fig. 7c, where the normalized spectra acquired under both excitation conditions are displayed. In the case of profile A, a shift of the maximum of the broad band from ~ 570 nm (~ 2.17 eV) to ~ 638 nm (~ 1.94 eV) is identified.

Interestingly, the same maxima were found for the composites prepared by pulsed electrodeposition, also exhibiting a similar spectral shape as the one of profile A. These results suggest that similar defect centers are formed in all cases, however with a slightly different defect concentration for the continuous process. Moreover, the recombination channels that give rise to emission at longer wavelengths seem to be promoted under higher excitation density conditions. It is also worth mentioning that the slight fluctuation in the intensity of the PL signal observed for the different samples is not significant and is likely due to small changes in the experimental optical alignment conditions and/or the quantity of ZnO rods present in the probed region.

The presence of broad bands in the visible spectral region is very frequent in ZnO crystals, both in bulk and in micro/nanostructures^57^. Indeed, the existence of orange/red luminescence bands has been widely reported in the literature^58,59^, especially in the case of low temperature synthesized materials^18^, and particularly in the ones prepared by electrodeposition^21,60,61^. The assignment of such optical transitions to a specific defect is not straightforward and several hypotheses have been raised in the literature. Independently of its origin, some reports^62,63^ propose that the formation of this orange/red emission band is promoted under oxygen-rich conditions. Djurisic et al.^58,64^ have claimed that an orange/red band is frequently observed in ZnO nanoneedles produced by thermal evaporation, whose intensity can be strongly affected by the annealing conditions, being enhanced in air ambiance, while inert atmospheres lead to its quenching. The most common origins suggested for the emissions occurring in this spectral region are the defects associated with an excess of oxygen, namely interstitial oxygen (O_i_), together with interstitial zinc (Zn_i_) or zinc vacancies (V_Zn_)^57^. In fact, the latter has been pointed out as a deep acceptor in this material, having the lowest formation energy among all native defects in n-type ZnO^63,65,66^. The connection between the ${V}_{Zn}^{-}$ and a red emission peaked at ~ 1.6 eV was suggested by Wang et al*.*^65^, while Lv et al.^67^ discussed that different charge states of such defect give rise to three different transitions peaked at ~ 414 nm (~ 2.99 eV), 525 nm (~ 2.36 eV), and 600 nm (~ 2.07 eV). Indeed, theoretical works indicated the V_Zn_ can be stable in five different charged states, which originate emission bands in the ultraviolet (~ 3.2 eV), green (~ 2.5 eV), and red (1.9–2.0 eV) regions^68^. The work conducted by Zubiaga et al*.*^69^ also evidenced that such defects are preferentially located near the ZnO surface. This is in line with previous works on ZnO structures prepared by the hydrothermal method^18^, which revealed a dependence of the PL intensity upon increasing photon illumination density as well as on the atmosphere (air *vs* vacuum) where the measurements were conducted. Indeed, this type of defects becomes more dominant in the case of nanostructures with a high aspect ratio^70^, as is the present case. As so, surface-related defects should also be accounted for the optical transitions observed in this region. As the bands identified in this work are very broad and comprise a wide spectral region from green to red, it is important to bear in mind that they are likely to be constituted by an overlap of multiple defect-related emissions, which results in their broad emission features. Thus, other common bands observed in ZnO peaked at the green and yellow regions^64^ may also be contributing to the observed emission. In particular, the appearance of the yellow luminescence is frequent in ZnO prepared by solution-based methods, and is typically associated with the presence of adsorbates at the surface of ZnO, namely OH groups^64,71^.

Besides the broad emission band, when probed with the He–Cd laser, the composites also exhibit the presence of the near band edge (NBE) emission in the UV region, although with a much lower intensity than the visible band (Fig. 7b). This emission is particularly evident when the composites are cooled down to 14 K (Fig. 7d). Even at low temperatures, the emission is dominated by the broad orange/red emission (see the picture in inset) in all cases, with just a small contribution from the NBE. For most of the composites, the NBE emission is peaked at ~ 367 nm (~ 3.378 eV), presenting an asymmetrical and broad spectral shape, likely associated with an overlap of the typical transitions that occur at this region, namely the free (FX) and bound (BX) excitons, surface excitons (SX) and defect-related transitions, as well as their phonon (LO) replicas^57,72^. In the case of profiles B and J, a well-defined line was identified at ~ 368 nm (~ 3.369 eV) and a smaller one at ~ 374 nm (~ 3.31 eV). While the first may be due to contribution from both FX and BX transitions, the latter has been associated with surface defects in ZnO micro/nanocrystals^73^.

Another important aspect to keep in mind when discussing the PL features of the present nanostructures is the presence of LIG in direct contact with the semiconductor crystals. For instance, ZnO/rGO composites have shown a decrease in the PL intensity, tentatively attributed to interfacial charge transfer between ZnO and rGO^74^. Similarly, combining ZnO and LIG produced simultaneously by direct laser scribing showed that when the ZnO structures were produced from metallic zinc, the PL spectra displayed a weak luminescence signal, comprised by an orange/red emission band and with a very small contribution from the NBE emission, comparable to the spectral features observed herein^33^. On the other hand, in the case of the ZnO/LIG composites prepared by mixing scraped LIG with ZnO produced by LAFD^31^, both the intensity and the spectral shape of the PL spectra were seen to change due to the addition of LIG. A higher amount of LIG in contact with ZnO led to an increase in the intensity of the excitonic transitions in the UV region, accompanied by a decrease in the intensity of the broad visible band (peaked in the green in that case). This behavior was attributed to both ZnO surface defect passivation and charge transfer between the two materials. Indeed, the interaction between carbon-based materials and ZnO is widely affected by the properties of each component, which may differ considerably depending on the type of structures and synthesis methods, resulting in subsequent variation in the alignment of the energy levels of both materials, namely the defect-related ones, leading to different luminescence features^33,75^.

**Figure 8:**
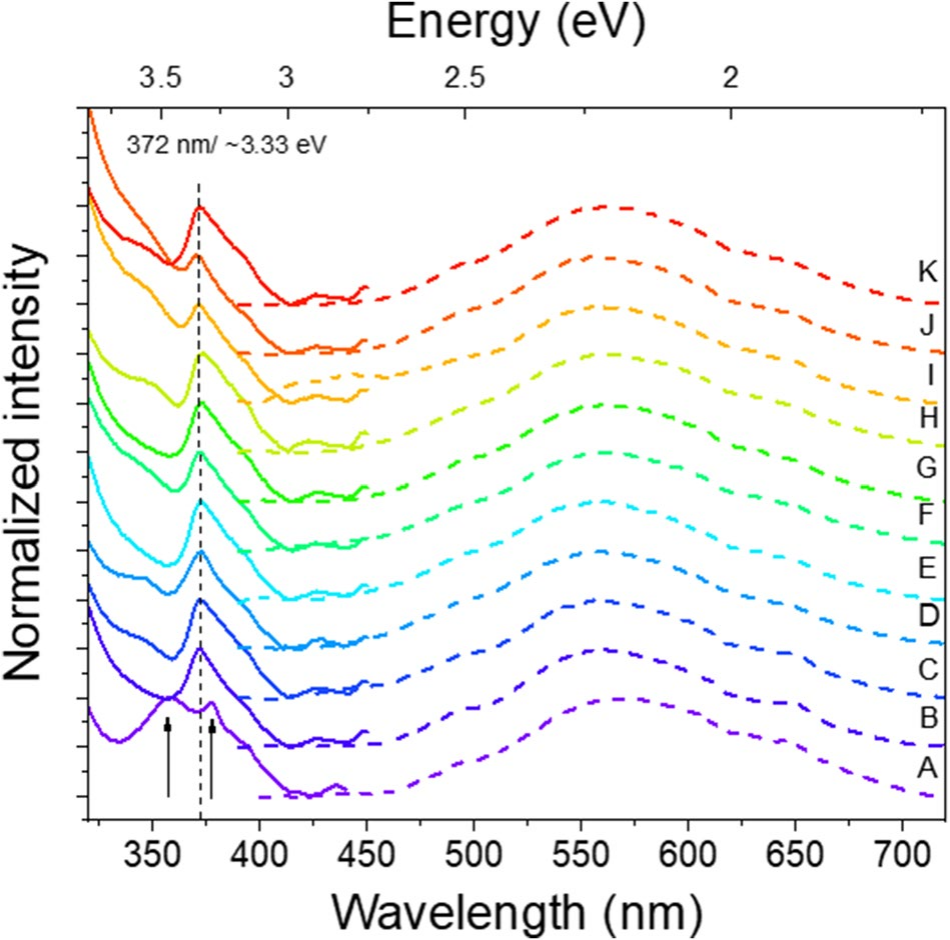
Normalized PL/PLE spectra of the LIG/ZnO composites. The spectra were vertically shifted for clarity. Solid lines: PLE @ 557 nm (peak of the orange band); Dash-dot lines: PL @ 325 nm (Xe lamp).

With exception of profile A, all composites show main excitation maxima at ~ 372 nm (~ 3.33 eV) in the PLE spectra (Fig. 8), which is fairly coincident with the expected bandgap energy of this semiconductor at RT. Besides this maximum, towards the higher energy (shorter wavelength) region, an increase in the spectra intensity was also observed. Such results indicate that the preferential population/excitation paths for the broad luminescence are via excitation with photons with energy equal to or higher than the ZnO bandgap. Additionally, looking at the lower energy (longer wavelength) region, all composites (including profile A) present an onset absorption near ~ 404 nm (~ 3.07 eV). The wide excitation tail that extends from that wavelength value towards the ZnO bandgap peak is likely associated with a wide distribution of defect states (e.g. surface-related) that create potential fluctuation in the semiconductor and lead to broadening of the impurity levels and a considerable density of states tail in the bandgap of the semiconductor. As so, a contribution from the density of states in this region should also be accounted in the population of the visible band too.

Contrarily, in the case of profile A, instead of the peak observed at ~ 372 nm, a broad excitation band is seen from ~ 330 nm (~ 3.76 eV) to ~ 420 nm (~ 2.95 eV). On the top of that broad band two peaks are clearly identifiable (black arrows in Fig. 8), one at ~ 359 nm (~ 3.45 eV) and another at ~ 378 nm (~ 3.28 eV). While the latter can be associated with the FX excitation, the former is well above the values that are expected for the ZnO bandgap. One possible explanation for this blueshift is the presence of a high concentration of impurities in this sample, which can give rise to the Burstein-Moss effect, typically observed in heavily-doped materials, resulting in a band filling that shifts the optical bandgap for higher energies^21,76,77^. However, if that was the case, a blueshift of the UV PL emission should be also observed, in line with what is verified in the above mentioned situation^21^. Yet, that was not observed in the here reported ones, as can be seen in Fig. 7d, where the peak position of the NBE emission of profile A is nearly the same as the remaining composites. Besides, the formation of additional non- stoichiometric ZnOx phases with higher bandgap energies may be promoted by the less controlled continuous deposition (profile A), which proceeds at a higher rate and in which the renewal of the diffusion layer is not promoted. For instance, such values of bandgap energy have been reported for the far less studied ZnO_2_ phase^78–80^. Previous work on ZnO/ZnO_2_ composites^80^ revealed a high-energy excitation band peaked at 362 nm (~ 3.42 eV) in the PLE spectrum that was attributed to the ZnO_2_ phase. This value is very close to the one obtained here (359 nm/~ 3.45 eV). It is important to note that despite ZnO_x_ phases other than ZnO wurtzite were not detected in the Raman and XRD measurements, small amounts could still be present below the respective limits of detection. As PL/PLE measurements are extremely sensitive, even residual amounts of optically-active species could still be observable. Therefore, we cannot exclude that other processes could be involved in the blue shift observed for the bandgap of the composite produced according to profile A conditions.

## Conclusions

Low temperature, simple and scalable production of foam-like multilayer graphene and ZnO composites is attained via electrodeposition of ZnO rods on LIG. Despite ZnO rods are produced by both continuous and pulsed electrodeposition, the latter allows for better growth control, yielding more faceted and regular hexagonal rods, at the cost of deposition rate. The uniform and conformal electrodeposition of ZnO takes place deep inside the LIG pores yet preserving its intricate pore network through which electrolyte can diffuse, as shown by EIS analysis employing transmission line models. To the best of our knowledge, this is the first time that a transmission line model that considers porosity is shown to be required to properly describe the impedimetric response of LIG and related composites, the common and simpler models based on the Randles equivalent circuits failing to describe the high-frequency portion of the impedance data.

PL studies reveal a broad orange/red luminescence band dominating the spectra of all composites when excited with 325 nm laser line, which is found to redshift when PL measurements are performed using the same excitation energy but at lower densities (employing a Xe lamp), undoubtedly showing that multiple recombination channels are involved. Moreover, for the composites produced by pulsed electrodeposition, the main PLE maximum was observed at ~ 3.33 eV, in line with the expected bandgap energy of ZnO at RT, contrasting with a broad excitation band recorded for the continuous profile that exhibits two peaks at ~ 3.45 eV and at ~ 3.28 eV, which can be associated to the presence of different types of defects and/or ZnO_x_ phases. The photoluminescence signal provided by the ZnO nanorods opens added possibilities in biosensing such as photoelectrochemical and/or simultaneous electrochemical and optical biodetection, triggering in situ counterproofing operation and extended/complementary detection ranges. In this sense, the composites are shown to provide swift electrochemical electron transfer, the rate constants reaching $1.24\times {10}^{-2}$ cm s^−1^ employing [Ru(NH_3_)_6_]^2+/3+^ redox probe, comparable to that of bare LIG. Hence, a proper LIG/ZnO intra composite electric contact is attained permitting an excellent charge transfer between LIG and ZnO, a crucial aspect in electrochemical and photoelectrochemical biodetection.

Notwithstanding, the role of ZnO rods goes beyond added optical functionality for biosensing. Compared to bare LIG, the composites show enhanced electrochemical capacitance up to ~ 1.4 mF cm^−2^ at 1 mA cm^−1^ in 1 M KCl, whilst maintaining low ohmic losses and excellent long term cycle stability. These results are a promising starting point towards envisaged advanced applications, such as all-solid-state flexible micro-supercapacitors employing gel polymer electrolytes.

## Methods

### Reagents

The PI sheets, precursors for LIG synthesis, consisted of Kapton® HN500 (127 µm thickness). Silver ink (Electrodag 1415) and non-reacting insulating varnish were supplied by Agar Scientific. Other employed reagents are Potassium hexacyanoferrate(II) trihydrate (K_4_[Fe(CN)_6_]·3H_2_O, > 99%), hexaamineruthenium(III) chloride ([Ru(NH_3_)_6_]Cl_3_, > 98%), potassium chloride (KCl, > 99.0%), Zinc nitrate hydrate (Zn(NO_3_)_2_·xH_2_O), 99% metals basis). Deionized (DI) water was obtained from a MilliQ water purification system, with a resistivity of 18.2 MΩ m^−1^. All reagents were used as received.

### LIG direct laser writing (DLW) synthesis

The DLW procedure was performed using a continuous CO_2_ laser equipped with a computer-driven gantry holding a focusing lens (Redsail M500). The scan is accomplished by the step motor driven translational motion of the laser focusing head in the *x* and *y* directions. Simplified schematics of the LIG DLW on PI are presented in Supplementary Fig. S1. The employed DLW parameters are summarized in Supplementary Table S1.

### Electrode assembly and ZnO electrodeposition

Electrical contacts were formed on LIG using copper wire and high-quality silver ink, baked at 130 °C for 5 min for improved mechanical strength and electrical conductivity. Afterwards, the contact insulation defining the geometrical active area of the electrodes, 0.81 cm^2^, was performed by coating with an inert varnish. After 24 h drying in air, the samples were washed repeatedly with isopropanol and DI water.

The electrodeposition apparatus comprised LIG, Zn plate and Ag/AgCl (1 M KCl, *CH Instruments*) as working, counter and reference electrodes, respectively. The electrolyte was 15 mM Zn(NO_3_)_2_·xH_2_O and 5 mM KCl in DI water. The cathodic reaction initiates with the reduction of nitrate, generating Zn ions for posterior ZnO deposition via intermediate zinc hydroxide formation and its subsequent oxidation^23^. The synthesis comprises a nucleation-promoting step, consisting of 10 cyclic voltammetry (CV) scans within 0.0 V to − 1.4 V at a potential scan rate of 0.1 V s^−1^ at 75 °C (Supplementary Fig. S2a). This way, ZnO nucleates on the LIG surface (Supplementary Fig. S2b) acting as seeds for further ZnO crystal growth. After seed deposition, the stirrer was turned on and the electrodeposition profiles were employed as described in Table 1 (also reproduced in Supplementary Table S2).

The pulsed profiles comprise deposition steps t_on_ at negative potentials V_on_ intercalated with resting steps t_off_ at 0 V (V_off_). For all samples, the total deposition time (i.e., the sum of t_on_ for all the pulses) was 750 s and the electrolyte temperature was 75 °C. Pulsed profiles are characterized by the duty cycle (t_on_/t_off_ in percentage) and frequency f (1/T, where T is the pulse period). Supplementary Fig. S3 further details a pulsed electrodeposition process.

### Morphological and structural characterization

The structural analysis of the LIG/ZnO samples was conducted via RT μ-Raman spectroscopy in backscattering configuration (Horiba Jobin–Yvon HR800 unit). The 442 nm laser line of a cw He–Cd laser (Kimmon IK series) was used as the excitation source, yielding a rich vibrational spectrum containing the signature of both graphene and ZnO at appropriate intensities. A Peltier-cooled (223 K) CCD detector and a 600 g mm^−1^ grating were employed and a 100× objective at 0.9 NA resulted in a spot size of ~ 1 μm. A neutral filter with optical density of 0.3 (< 5mW at the sample) was used to avoid overheating, while all spectra were acquired with an exposure time of 5 s and 6 accumulations. All spectra were corrected to the background and the offset of the edge filter at lower frequencies. Scanning electron microscopy (SEM) measurements were performed via a TESCAN-Vega3 SBH SEM unit. Selected samples were also subjected to X-ray diffraction (XRD) measurements using a PANalytical X'Pert PRO diffractometer operating in the Bragg–Brentano configuration and with CuKα radiation (λ = 1.54056 Å).

### Electrochemical measurements

The electrochemical cell was set up in high solution volume (75 mL) to electrode active area (0.81 cm^2^) ratio conditions. A three-electrode configuration was used, where the LIG/ZnO composites, a platinum wire, and an Ag/AgCl (1 M KCl) constituted the working, counter, and reference electrodes, respectively. Cyclic voltammetry, galvanostatic charge–discharge (GCD) curves and electrochemical impedance spectroscopy (EIS) data were acquired via a *Versastat3* (Princeton Applied Research) unit. Prior to experiments, the electrolyte solution was stirred and bubbled with N_2_ for 30 min and kept under N_2_ blanket. EIS measurements were performed applying a 10 mV AC perturbation at a DC bias of 0 V versus Ag/AgCl, acquiring a logarithmic distribution of 10 points per decade in the 1 kHz to 10 mHz range. Models were constructed via *E-Chem Analyst* Software from *Gamry* and fittings were performed using the built-in Simplex algorithm. GCD curves were recorded at a varying current density between 0 and 0.8 V vs. Ag/AgCl.

The areal capacitance (C) of the electrodes were calculated from the GCD curves using the following relation^81^,

1 $$\text{C}= \frac{{\text{It}}}{{\text{U}}},$$

where I is the current density and $\text{t}$ and $\text{U}$ are the discharge time and voltage limits, respectively.

The heterogeneous electron transfer standard rate constants (${\text{k}}_{\text{eff}}^{0}$) were derived from cyclic voltammograms (CV) in 0.1 M KCl and 0.5 mM [Ru(NH_3_)_6_]^3+^ solutions employing the Nicholson method^51^. A dimensionless kinetic function ($\Psi$) is constructed via the following relation^54^:

2 $$\Psi =\frac{-\,0.6288\,+\,0.0021\Delta {\text{E}}_{\text{p}}(\nu) }{1-0.017\Delta {\text{E}}_{\text{p}}(\nu)} ,$$

where $\nu$ is the potential scan rate and $\Delta {\text{E}}_{\text{p}}(\nu)$ is the scan rate dependent potential difference between the anodic and cathodic peaks of the cyclic voltammograms. The ${\text{k}}_{\text{eff}}^{0}$ is then retrievable via the slope of the fitting of the linear portion of the $\Psi (\nu)$ function^51^.

3 $$\Psi =\frac{{\text{k}}_{\text{eff}}^{0}}{{\left(\frac{{\text{n}}\pi {\text{FD}{\nu}}}{\text{RT}}\right)}^\frac{1}{2}},$$

in which n is the number of electrons involved in the redox reaction, D is the diffusion coefficient for [Ru(NH_3_)_6_]^2+/3+^ in KCl aqueous solution (9.1 × 10^−6^ cm^2^ s^−1^^54^), F is the Faraday constant (96,485.33 C mol^−1^), R the universal gas constant (8.314 J mol^−1^ K^−1^) and T the absolute temperature (K).

### Photoluminescence and photoluminescence excitation spectroscopies

All samples were analyzed by steady-state photoluminescence (PL) and PL excitation (PLE) spectroscopies at RT. The experiments were conducted in a Fluorolog-3 Horiba Scientific set-up with a double additive grating Gemini 180 monochromator (1200 g mm^−1^ and 2 × 180 mm) in the excitation and a triple grating iHR550 spectrometer in the emission (1200 g mm^−1^ and 550 mm). A 450 W Xe lamp was used as the excitation source and the excitation monochromator was fixed at 325 nm. The PLE was measured by setting the monochromator in the energy position of the emission maxima of interest and, afterward, the excitation was scanned for higher energies. Additionally, PL spectra for selected samples were also acquired both at RT and low temperature (14 K) by exciting them with the 325 nm (~ 3.81 eV) line of a Kimmon cw He-Cd laser (power density I_0_ < 0.6 W cm^−2^, superior to the Xe lamp). In these measurements, the luminescence radiation was dispersed by a SPEX 1704 monochromator (1 m, 1200 g mm^−1^) and detected with a cooled Hamamatsu R928 photomultiplier. The 14 K PL studies were carried out by placing the samples on a cold finger He cryostat.

## Supplementary Information

Supplementary Information.

## Data Availability

The datasets generated during and/or analysed during the current study are available from the corresponding author on reasonable request.
