# Supplementary material for: Electrochemical and photoluminescence response of laser-induced graphene/electrodeposited ZnO composites
Source: Sci Rep. 2021 Aug 25;11:17154. doi: 10.1038/s41598-021-96305-8 (PMC8387487; doi:10.1038/s41598-021-96305-8)
Supplement: Supplementary file 1 — Supplementary Information. [file 41598_2021_96305_MOESM1_ESM.pdf]

Supplementary Information

Electrochemical and Photoluminescence Response of Laser-induced Graphene/Electrodeposited ZnO Composites

N. F. Santos*, J. Rodrigues, S. O. Pereira, A. J. S. Fernandes, T. Monteiro, F. M. Costa

I3N, Department of Physics, University of Aveiro, Campus de Santiago, 3810-193 Aveiro, Portugal

*Corresponding author: nfsantos@ua.pt

1. Methods

Fig. S1 shows a simplified schematics of the direct laser writing (DLW) of graphene on polyimide (Kapton®) foils. **Table S1** gathers the employed laser writing parameters for LIG production, which served as electrodes for ZnO electrodeposition.

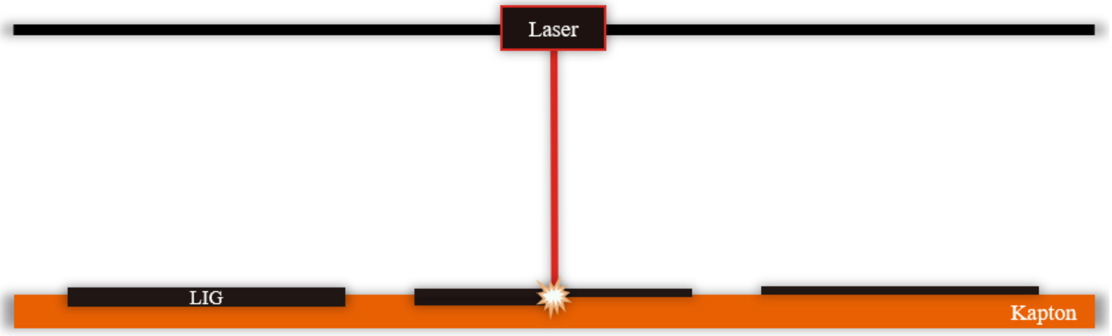

Fig. S1: Simplified schematics of LIG DLW synthesis.

Table S1: Employed parameters for LIG DLW synthesis.

**PI foil thickness**	75 μm
**Laser wavelength**	10.6 μm
**Scan type**	Unidirectional
**Laser scan rate**	250 $mm.s^{-1}$
**Measured laser power**	12.5 W
**Line-to-line distance**	75 μm
**Laser head-to-foil distance**	19.8 mm
**Distance from focus**	1.8 mm (above focus)

Fig. S2a shows a CV cycle for ZnO seed formation on LIG. The seeds deposition occurs during the negative (forward) scan. During the scan down to -1.4 V vs. Ag/AgCl (1 M KCl), reduction of nitrate initiates at about -0.8 V vs. Ag/AgCl (1 M KCl), freeing Zn ions for posterior ZnO deposition via intermediate zinc hydroxide formation and subsequent oxidation, culminating in ZnO seeds on the LIG surface[1]. During the positive (reverse) scan no relevant faradaic activity is seen, meaning that the growing ZnO deposits are stable[1]. **Fig. S2b** shows SEM images of the deposited ZnO seeds on LIG.

After seed deposition, the electrodeposition profiles presented in **Table S2** were employed. **Fig. S3** schematizes a pulsed electrodeposition process (profile B). The pulsed profiles comprise deposition steps t_{on} at negative potentials V_{on} intercalated with resting steps t_{off} at 0 V (V_{off}). For all samples, the total deposition time (i.e., the sum of ton for all the pulses) was 750 s and the electrolyte temperature was 75 ºC. Pulsed profiles are characterized by the duty cycle (t_{on}/t_{off} in percentage) and frequency f (1/T, where T is the pulse period).

As relevant aspects of the employed electrodeposition process, it is to note that the use of zinc nitrate has advantages over other types of precursors as it provides both zinc and oxygen species without the need for additional oxidants. Moreover, the electrodeposition was performed using high surface area zinc counter electrodes instead of platinum or other inert electrodes, guaranteeing that Zn ions are created at the counter electrode compensating the consumed Zn on the LIG (working) electrode. This prevents zinc exhaustion and consequent decrease in pH due to H^+ ion buildup at an inert counter electrode, known to hinder the growth rate and quality of the ZnO deposits[2].

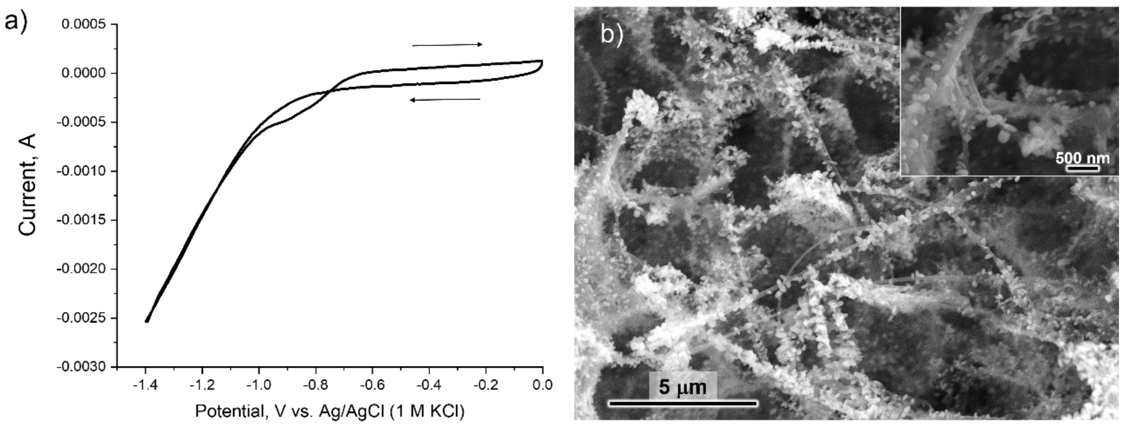

Fig. S2: **a)** Cyclic voltammogram (5th cycle) for seed layer formation on LIG. The scan rate is 0.1 $V.s^{-1}$. **b)** SEM images of seeds on LIG after 10 CV cycles.

Table S2: Employed parameters for ZnO pulsed electrodeposition on LIG. A continuous electrodeposition was also performed for comparison purposes.

Profile	t_{on} (s)	t_{off} (s)	Duty cycle (%)	V_{on} (V vs. Ag/AgCl, 1 M KCl)	Frequency, f (Hz)	Number of pulses
A (Continuous)	-	-	-	- 1.15	-	-
B	0.5	1.5	25	- 1.15	0.5	1500
C	1	1	50	- 1.15	0.5	750
D	1.5	0.5	75	- 1.15	0.5	500
E	0.25	1.75	12.5	-1.15	0.5	3000
F	0.5	1.5	25	-1.05	0.5	1500
G	0.5	1.5	25	-1.25	0.5	1500
H	0.5	1.5	25	-1.35	0.5	1500
I	0.05	0.15	25	-1.15	5	15000
J	0.005	0.015	25	-1.15	50	150000
K	5	15	25	-1.15	0.05	150

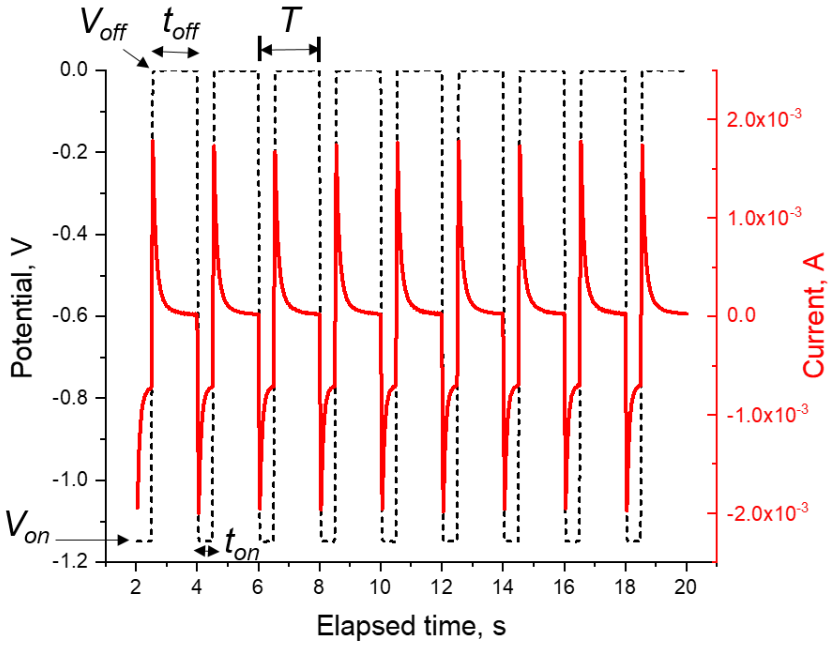

Fig. S3 - Potential (black dotted lines) and current profiles (solid red line) for electrodeposition profile B (2nd to 10th pulses).

2. Morphological and structural analysis

Fig. S4 shows SEM images of electrodeposited ZnO for the remaining profiles not shown in **Fig. 1** of the main manuscript, along the respective rod width distribution in the bottom left insets.

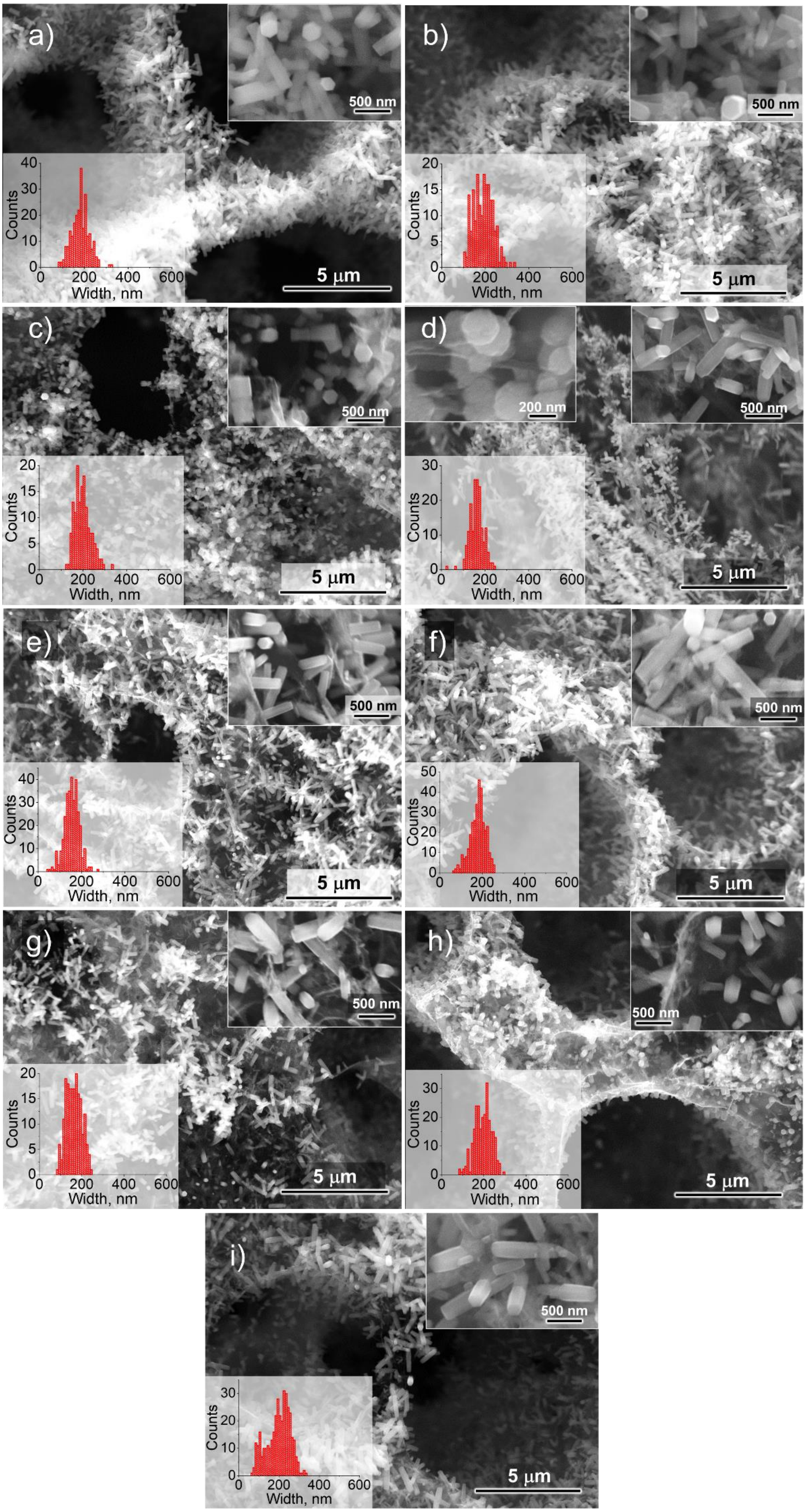

Fig. S4: Top-view SEM images and statistical analysis of ZnO rod width for pulsed profile **a)** C, **b)** D, **c)** E, **d)** F, **e)** G, **f)** H, **g)** I, **h)** J and **i)** K.

Fig. S5 shows the Raman spectra of LIG/ZnO composites produced via the remaining electrodeposition profiles not shown in **Fig. 3** of the main manuscript (see also **Table S2** for synthesis parameters).

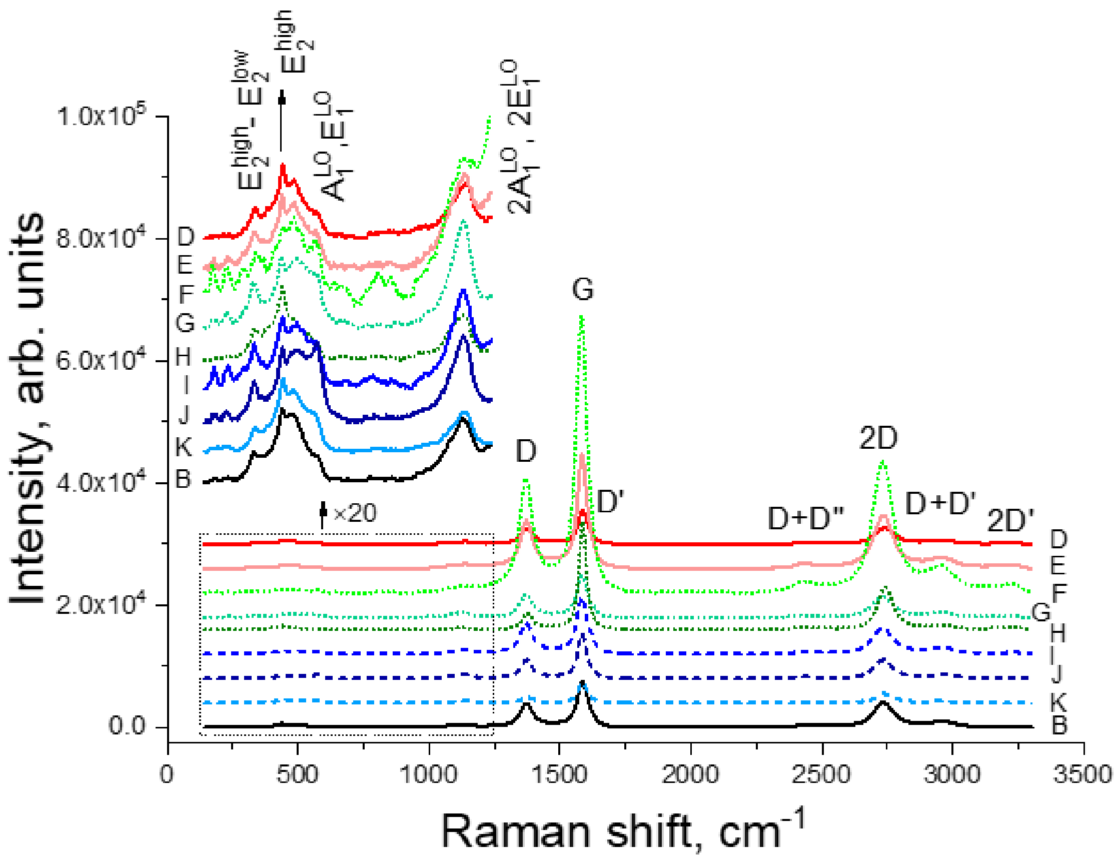

Fig. S5: Background-subtracted μ-Raman spectra at 442 nm photon excitation of the LIG/ZnO composites produced by pulsed electrodeposition (profiles B, D, E, F, G, H, I, J and K as listed in **Table S2**). The spectra are normalized to the peak intensity of the E_2^{high} vibrational mode of ZnO (~439 cm^{-1}) and shifted in intensity for clarity.

In **Fig. S6** are depicted the X-ray diffractograms of the polyimide (PI) substrate, LIG as-grown and LIG/ZnO composites synthesized employing electrodeposition profiles A (continuous) and B (pulsed).

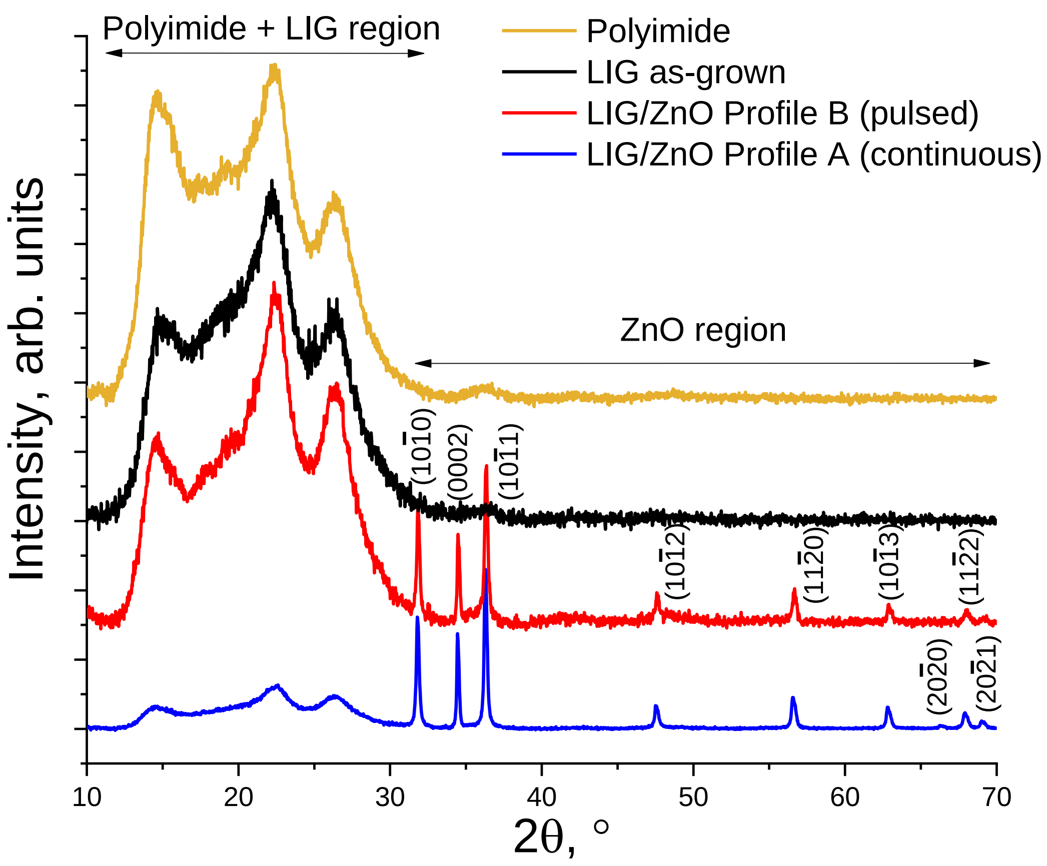

Fig. S6: Backgroud-subtracted X-ray diffractograms of the PI substrate, LIG as-grown and LIG/ZnO (profile A) and LIG/ZnO (profile B) composites. For comparison purposes, the diffractograms of the composites were normalized to the ZnO (wurtzite) $(10\bar{1}1)$ peak intensity, whereas the diffractograms of the LIG as-grown and PI samples were normalized to the the LIG/ZnO (profile A) diffractogram maximum at $2\theta \cong$ 22.4°.

Regarding the ZnO phases, both pulsed (profile B) and continuous (profile A) samples' diffractograms are fully described by the ZnO (wurtzite) reflections through ICDD database code 04-021-6114. Both profiles denote narrow ZnO (wurtzite) peaks with similar full widths at half maximum, *e.g.* $\Delta\,(2\theta) \cong 0.24°$ for the $(10\bar{1}1)$ reflection, showing similar (high) crystallinity for the two samples. The PI foil diffractogram displays the typical PI signature [3,4] with broad peaks at $2\theta \cong$ 14.8°, 22.4° and 26.5°. This diffraction region superimposes with that expected for the (0002) reflections from graphene and graphene-derivatives. Hence, for the samples containing LIG, this region is likely a linear combination of contributions from PI and LIG. This might explain the prominence of the reflections at $2\theta \cong$ 22.4° and 26.5° for all LIG-containing samples compared to the PI foil diffractogram. The difference in the relative intensities of the PI/LIG-related reflections for the two composite samples are due to the lengthier and more agglomerated ZnO rods produced via the continuous electrodeposition profile A, as

seen in **Fig. 1** and **Fig. 2** of the main manuscript. This results in a thicker and denser ZnO layer that attenuates the X-ray intensity reaching the PI/LIG backbone, in analogue to the observed in the Raman spectra of the composites (**Fig. 3** of the manuscript).

3. Electrochemical measurements

Fig. S7 depicts the galvanostatic charge-discharge cycle stability of the LIG/ZnO (profile C) electrode up to 5000 cycles between 0 and 0.8 V vs. Ag/AgCl (1 M KCl) at 1 mA.cm^{-2}. **Fig. S8** shows the Bode and high-frequency Nyquist plots of the bare LIG and LIG/ZnO (profile A) electrodes, along the fittings employing the three different models considered in this work, the modified Randles (MR), Bisquert open (BTO) and modified unified Bisquert (MUB). Please refer to **Fig. 5** of the main manuscript for model schematics and related text for model description.

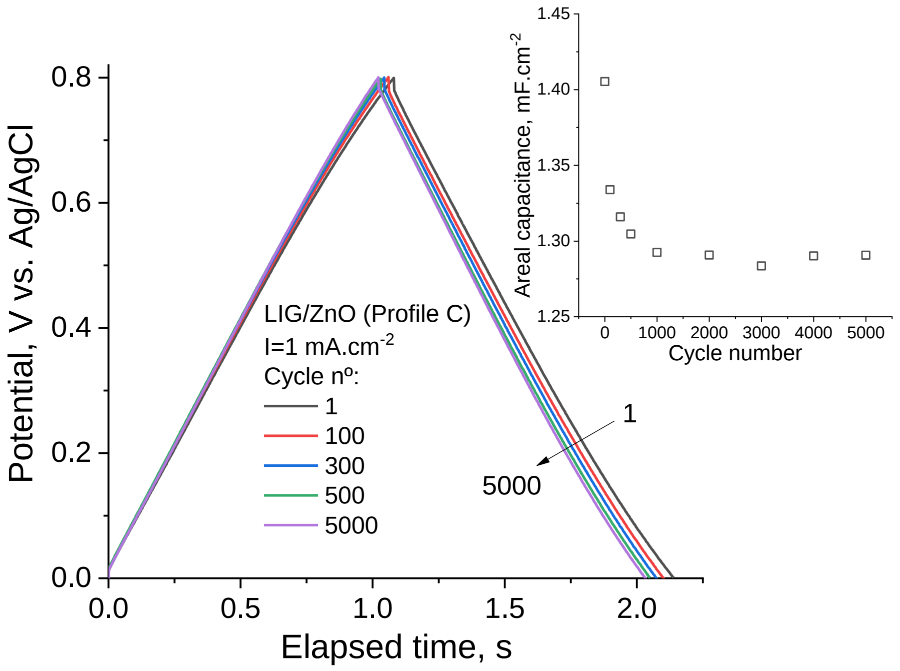

Fig. S7: Endurance stability of GCD curves for the LIG/ZnO (profile C) electrode. Inset shows derived capacitance evolution with cycle number.

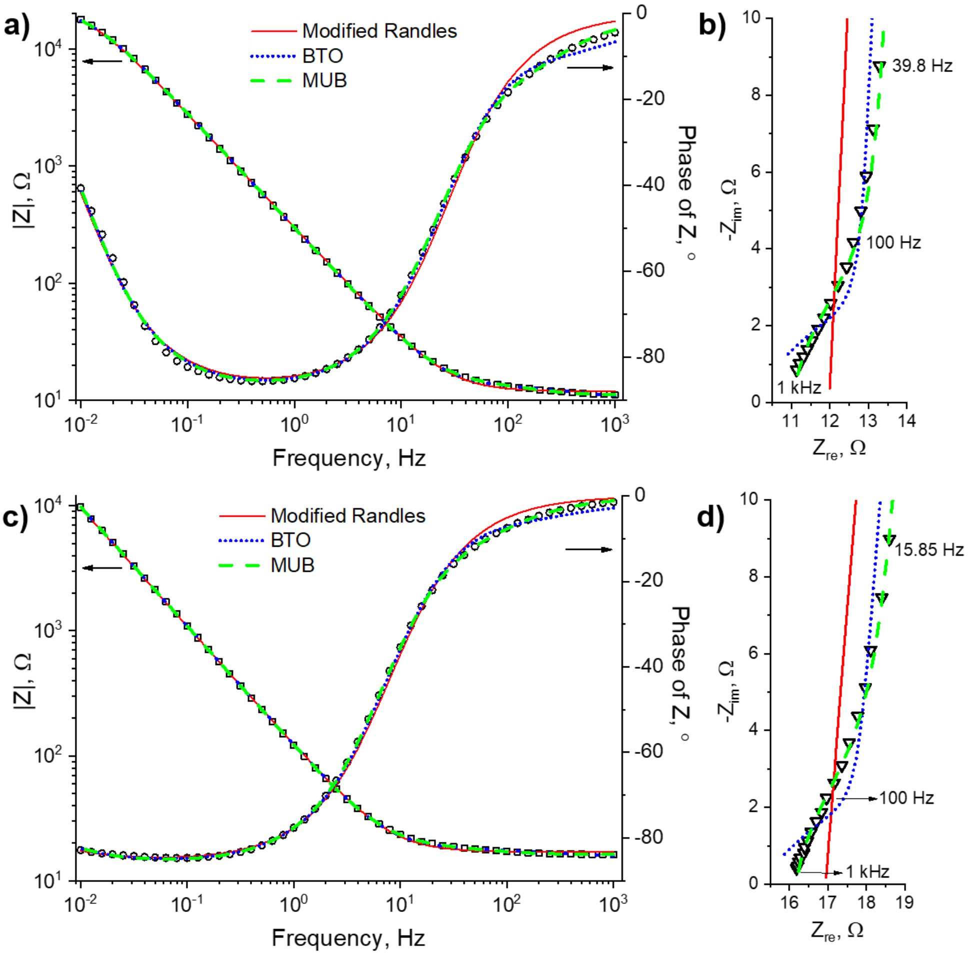

Fig. S8: Bode and high-frequency Nyquist plots for the (**a** and **b**) bare LIG and (**c** and **d**) LIG/ZnO (Profile A) electrodes, respectively. Equivalent circuit fittings employing the MR, BTO and MUB models are also shown. The electrolyte is 1 M KCl aqueous solution.

Table S3, **Table S4** and **Table S5** list the fitting parameter values of the bare LIG and LIG/ZnO (profile A) electrodes using the MR, BTO and MUB models, respectively.

Table S3: Parameter values of EIS fittings using the modified Randles model (i).

Sample	R_s (Ω)	R_{LEAK} (Ω)	P_0* ($s^a.\Omega^{-1}$)	a*
**LIG**	12.0	2.45×10^{4}	5.60×10^{-4}	0.970
**LIG/ZnO (Profile A)**	16.9	2.20×10^{5}	1.43×10^{-3}	0.950
**LIG/ZnO (Profile C)**	13.2	6.48×10^{4}	1.41×10^{-3}	0.950

$^*Z_Q = \frac{1}{P_0\,(iw)^a}$, where i is the imaginary unit and w is the angular frequency.

Table S4: Parameter values of EIS fittings using the Bisquert open (BTO) model (ii).

Sample	R_s (Ω)	R_{EL} (Ω)	R_{PORE} (Ω)	$P_{0,PORE}$* ($s^a.\Omega^{-1}$)	a_{PORE}*
**LIG**	9.60	9.46	2.40×10^{4}	5.58×10^{-4}	0.977
**LIG/ZnO (Profile A)**	15.0	7.95	1.66×10^{5}	1.43×10^{-3}	0.955
**LIG/ZnO (Profile C)**	11.3	8.40	5.70×10^{4}	1.41×10^{-3}	0.957

$^{*}Z_Q = \frac{1}{P_0\,(iw)^a}$, where ***i*** is the imaginary unit and ***w*** is the angular frequency.

Table S5: Parameter values of EIS fittings using the modified unified Bisquert (MUB) model (iii).

Sample	R_s (Ω)	R_{EL} (Ω)	R_{PORE} (Ω)	$P_{0,PORE}$* ($s^a.\Omega^{-1}$)	a_{PORE}*	$P_{0,BASE}$* ($s^a.\Omega^{-1}$)	a_{BASE}*
**LIG**	11.0	15.85	2.39×10^{4}	3.73×10^{-4}	0.972	1.85×10^{-4}	0.992
**LIG/ZnO (profile A)**	16.2	16.48	1.50×10^{5}	9.15×10^{-4}	0.946	5.19×10^{-4}	0.979
**LIG/ZnO (profile C)**	12.5	16.45	5.44×10^{4}	9.23×10^{-4}	0.953	4.90×10^{-4}	0.975

$^{*}Z_Q = \frac{1}{P_0\,(iw)^a}$, where i is the imaginary unit and w is the angular frequency.

Fig. S9 shows the scan rate (ν) dependent cyclic voltammograms of the LIG/ZnO (profile C) electrode using the $[FeCN_6]^{3-/4-}$ redox probe. The $\log(I_P)$-$\log(\nu)$ and I_P -ν dependencies are also shown. Note the linear I_P -ν relationship arising from adsorption and/or limitations to the semi-infinite diffusion regime due to the thin layer effect.

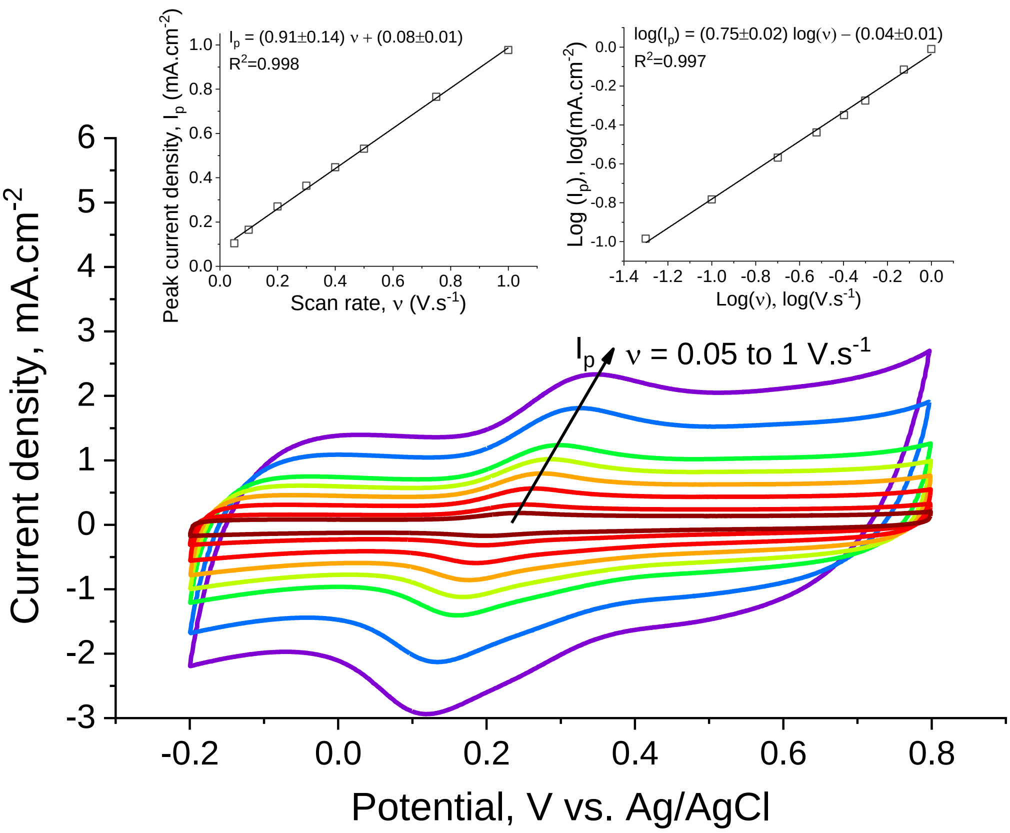

Fig. S9: Cyclic voltammograms at varying scan rate in 0.1 M KCl plus 0.5 mM $[FeCN_6]^{4-}$ aqueous solution for the LIG/ZnO (Profile C) electrode. The I_P -ν and log(I_P)-log(ν) plots are also shown in the left and right insets, respectively, along pertinent fittings.

References

1. Mahalingam, T., John, V. S., Raja, M., Su, Y. K. & Sebastian, P. J. Electrodeposition and characterization of transparent ZnO thin films. *Sol. Energy Mater. Sol. Cells* **88**, 227–235 (2005).
2. Sielmann, C., Walus, K. & Stoeber, B. Zinc exhaustion in ZnO electrodeposition. *Thin Solid Films* **592**, 76–80 (2015).
3. Qi, H., Qian, Y., Xu, J., Zuo, J. & Li, M. An AZ31 magnesium alloy coating for protecting polyimide from erosion-corrosion by atomic oxygen. *Corros. Sci.* **138**, 170–177 (2018).
4. Demirci, U. B. & Miele, P. Cobalt in NaBH4 hydrolysis. *Phys. Chem. Chem. Phys.* **12**, 14651–14665 (2010).
